# Response to Detection of New Delhi Metallo-β-Lactamase–Producing Bacteria, Brazil

**DOI:** 10.3201/eid2106.140113

**Published:** 2015-06

**Authors:** Leonardo Neves Andrade, Ana Lúcia Costa Darini

**Affiliations:** Universidade de São Paulo, Faculdade de Ciências Farmacêuticas de Ribeirão Preto, Ribeirão Preto, São Paulo, Brazil

**Keywords:** New Delhi metallo-beta-lactamase, New Delhi metallo-β-lactamase, MBL, NDM, carbapenemase, carbapenem resistance, bacteria, antimicrobial resistance, dissemination

**To the Editor:** New Delhi metallo-β-lactamase (NDM) is an example of a successful antimicrobial drug resistance determinant and has become one of the most clinically significant carbapenemases. The gene *bla*_NDM_ was first described in India in 2009. Its dispersion is epidemiologically linked to the Indian subcontinent, from which increased international transmission has been detected in nosocomial, community, and environmental isolates (*1*). Currently, the main acquired carbapenemases around the world are *Klebsiella pneumoniae* carbapenemase (KPC), oxacillinase-48 (OXA-48), and NDM. KPC is broadly detected and endemic to some areas; OXA-48 has been widely disseminated throughout European countries and has been reported in other regions. NDM is reported almost worldwide but did not successfully spread in most countries of Europe except the United Kingdom and recently, France, as has been found in *Enterobacteriaceae* (*2*) and in nonfermenting gram-negative bacilli, with progression toward rapid global prevalence.

NDM producers were detected most recently in South America (*3*). However, an increase in cases of NDM-producing bacteria has been noted. Carvalho-Assef et al. (*4*) described characterization of NDM in Brazil, in *Providencia rettgeri* isolated from a tissue sample excised from a patient in a hospital in Rio Grande do Sul state in southern Brazil, in 2013. Reports by Carvalho-Assef et al. (*5*) and Rozales et al. (*6*) have highlighted that *P. rettgeri* and isolates from the *Enterobacter cloacae* complex, clonally and nonclonally related, have been increasingly detected in the southern region of Brazil. In addition, retrospective studies have shown that NDM-1–producing *Enterobacter* have been present in Brazil since 2012 and have also been detected in Rio Grande do Sul (*5*). NDM-1–producing *Morganella morganii* (*6*), *Escherichia coli*, *Klebsiella pneumoniae* (*7*), *Acinetobacter baumannii* (*8*), and *Citrobacter freundii* (J. Campos, et al., https://www.escmid.org/escmid_library/online_lecture_library/?search=1&current_page=1&search_term=Citrobacter+freundii+NDM) have also been reported. Initial reports from Brazil also indicate that NDM producers have displayed characteristics such as co-resistance (*5*,*9*,*10*) and heteroresistance (*11*), but to date, occurrence in the community has not been reported. NDM producers were originally detected in the southern and southeastern regions of Brazil and have since moved into the northern states.

Brazil is a country of extremes that has industrialized and nonindustrialized regions, and this situation converges with social, economic, and infrastructure problems (e.g., sanitation and health care public services). This scenario is similar to the initial conditions that contributed to worldwide dissemination of NDM from the Indian subcontinent. Successful and widespread international high-risk clones and epidemic plasmids have been detected in Brazil and could have a critical role in rapid national expansion of NDM-encoding genes and NDM producers. Brazil is under imminent threat of national spread and prevalence of NDM.

Lessons learned from management of KPC-production bacteria will help infection prevention and control teams, clinicians, and microbiology laboratories in Brazil more effectively manage patients with infections caused by NDM producers. Moreover, because of knowledge gained associated with global KPC dissemination, including in Brazil, this country is better prepared to face the other β-lactamases emerging worldwide. Initiatives of regional agencies and the Brazilian Health Surveillance Agency include dissemination of risk alerts to health professionals and guidelines for technical standardization and management of multidrug-resistant bacterial infection. These alerts focus on NDM producers and specific prevention and control measures; therapeutic orientation; and interpretive criteria for the assessment of bacterial antimicrobial drug susceptibility. These efforts are aimed at rapid microbiology detection and clinical and epidemiologic measures to control infections caused by NDM producers and *bla*_NDM_ dissemination (e.g., using simple tests to detect carbapenemases, searching for colonized patients, and evaluating/monitoring hospital discharge of patients after NDM infection).

Another type of mobilization was promoted during the 27^th^ Brazilian Congress of Microbiology, 29 September–3 October, 2013 in Natal, Brazil, in which a special symposium was dedicated to discussing and improving NDM counterattacks ( http://www.sigeventos.com.br/sbmicrobiologia/admin/pro_lista_programa.asp?eveId=5&tipId=22 [in Portugese]). This symposium gathered a team of experts, including government representatives, to try to minimize the spread and effect of the entry of NDM into Brazil, as well as the future complications and consequences of national dissemination 

These efforts are crucial and have strategic significance that affect major events in Brazil, such as the 2014 FIFA World Cup, which brought people from all around the world to all regions of Brazil, and the upcoming Games of the XXXI Olympiad, which will occur in Rio de Janeiro in August 2016. Also of importance to this issue are the geopolitical and economic characteristics of Brazil. Because of Brazil’s global influence related to national and international movement of people and products, broad prevalence of NDM producers in Brazil could contribute to acceleration of the global spread of *bla*_NDM_. Microbiologists, clinicians, and their respective organizations, as well as the government of Brazil, through its health and disease control agencies, should continue to strive to contain NDM dispersion and limit the possible global impact of the spread and prevalence of NDM producers in Brazil.
